# Epidemiology of suicide in the Tri-City metropolitan area in Poland in 2010–2019

**DOI:** 10.1007/s00406-022-01548-9

**Published:** 2022-12-30

**Authors:** Karol Karnecki, Tomasz Gos, Johann Steiner, Dobrosław Mańkowski, Michał Kaliszan

**Affiliations:** 1 Department of Forensic Medicine, Faculty of Medicine, Medical University of Gdańsk, 23 Dębowa St, 80-204 Gdańsk, Poland; 2 Department of Psychiatry, Otto-Von-Guericke University, 44 Leipziger St, 39120 Magdeburg, Germany; 3 Center for Behavioral Brain Sciences (CBBS), Otto-Von-Guericke University, Universitätsplatz 2, 39106 Magdeburg, Germany; 4 Institute of Sociology, Faculty of Social Sciences, University of Gdańsk, Gdańsk, Poland

**Keywords:** Suicide, Epidemiologic studies, Community psychiatry, Alcoholism, Forensic medicine

## Abstract

The paper, which is a continuation of our previous epidemiological studies on the phenomenon of suicide in the Tri-City metropolitan area, presents the results of statistical analyses of suicides in the autopsy material of the Department of Forensic Medicine of the Medical University of Gdańsk in the years 2010–2019. The purpose of the study was to analyse in detail demographic data of suicides (age, sex, place of death), as well as to assess suicide methods and the impact of alcohol on suicides in the study area. During the 10-year study period, 8495 autopsies were performed, of which 1261 were suicides (14.8%). Statistical analyses were conducted using the statistical data analysis software system STATISTICA, version 13 (StatSoft, Tulsa, Oklahoma, USA). The results of the study indicate a continuing downward trend in the number of suicides since the beginning of the 21th century, with the number of suicides in rural areas increasing over the same period. In the analysed cohort, suicides were committed in particular by middle-aged men and the number of suicides among older people (65 +) increased at the same time. The increase in suicide occurred in late autumn and early spring. The most common method of suicide was hanging. There was a high percentage of inebriated victims (45%), and a comparison of the present studies with previous ones indicates the increasing impact of alcohol on suicide.

## Introduction

Suicide is one of the leading causes of death in the world. Globally, almost 800,000 people die of suicide every year [1] and 80% of suicides are committed in low and middle-income countries [2]. The WHO states that in 2019 the global age-standardised suicide rate was 9.0 per 100,000 population; however, suicide rates range from less than 5 per 100,000 across North Africa, the Middle East and some Mediterranean countries (e.g., Greece) to approximately over 20 per 100,000 across Eastern Europe, South Korea, Zimbabwe, Guyana and Suriname [3].

In spite of a decreasing trend in suicide rates since 1980, which was particularly accentuated in the past 20 years when the global suicide rate dropped by 36% [2, 4], there are still regions (the Americas) where the number of suicides is increasing [2]. Suicide, in particular, is highlighted as a problem in teenagers and young adults in the 15–29 age range, where it constitutes the 3rd and 2nd cause of death, respectively [5]. However, a comparison of age-adjusted rates indicates that older people (70 +) are the most exposed to death by suicide as suicide rates in that group are 8 times higher than among younger people [1, 6]. There are many exceptions to the above rule, e.g. high suicide rates among young adults in low-income countries [7] or high (the highest) suicide rates in the age group of 45–64 years, in addition to 85 years and above, including the USA and Poland [8, 9].

The WHO data reflect that in 2019 in Poland the suicide mortality rate was 11.3 per 100,000, including in particular a high suicide rate among men (20.1 per 100,000) and a much lower suicide rate among women (3.1 per 100,000) [2]. Globally, the male-to-female suicide ratio is 2.3, which means that suicides are committed twice as often by men than by women. However, in most countries, the male-to-female ratio is much higher, e.g., in Ghana (10.52), St. Vincent and the Grenadines (7.98), as well as in Poland (7.44), which is in 8th place in this classification in the document of 2017 [10]. Ratios below 1.0, which reflect higher suicide rates among women than men, are only recorded in Morocco (0.96). In two other countries, they are close to 1.0—Pakistan (1.02) and Bangladesh (1.09) [10]. On average, in high-income countries, male-to-female suicide ratios are over 3, while in low-income and middle-income countries, they are below 3, which means that suicides committed by women have a greater share in the total suicide group in those countries [2].

The existing literature, including the official WHO data, presents many dependencies between the suicide method and various factors, including demographic or class factors, and the availability and lethality of a given method. Particularly visible differences in suicide methods are observed among countries or regions. Thus, hanging is the most common method of suicide in most countries, with the highest percentage (up to 90%) in Eastern European countries, including Poland. In turn, in the US and Argentina, Switzerland and Uruguay, firearm suicides are the most common. Jumping from the height plays an important role in smaller urbanised countries, including Hong Kong, Luxembourg and Malta. In turn, poisoning with pesticides as a suicide method is a major problem in rural countries in Latin and South America, Asia, but also Portugal [11].

It is generally accepted that the majority of completed suicides (98%) have at least one mental disorder, most often depression, substance-related disorders (in particular alcohol use disorder), schizophrenia, and personality disorders [12]. The first epidemiological study of the mental condition of Polish population, which was published in 2012 (“EZOP I”), indicates that 23.4% of Poles can be diagnosed with at least one of mental disorders defined in the ICD-10 and DSM-IV classification systems, representing about 6 million Poles aged 18–64. The most frequent diagnoses included: substance use disorders (12.8%), including alcohol use disorder (abuse and dependence) (11.9%), neurotic disorders (9.6%) and anxiety disorders (3.5%). Moreover, important risk factors proximal to suicide exacerbate mental disorders by accentuated stress reaction, which plays also an independent role predisposing to suicide. Among them are factors with profound social and economic implications, like an unemployment with disproportions and abrupt changes in incomes, often associated with periods of economic recession [5, 13]. Of particular importance is social inequality, which amplifies suicide rates mainly in lower income groups, regardless of country income classification [5].

## Materials and methods

This paper, which is a continuation of studies we conducted on the phenomenon of suicide in the years 1980–2009 [14], presents the results of an analysis of full post-mortem autopsies of suicides in the years 2010–2019 in the Tri-City in northern Poland, consisting of three contiguous coastal cities (Gdańsk, Gdynia, Sopot) and the neighbouring Gdańsk County, encompassing a population of 865,185 (31 December 2019). In the analysed 10-year period, 8495 autopsies were performed, including 1261 suicides (14.8%).

Apart from the analysis of changes in the number (rate) of suicides, including so-called seasonality, we also assessed demographic data of suicides, including age, sex and place of death, categorised into suicides committed in the urban area and in the rural area. Suicides classified as “urban area” were suicides committed in four cities: Gdańsk, Gdynia, Sopot and Pruszcz Gdański, the only town in the Gdańsk County (area: 431.31 km^2^, population: 780,312), while suicides classified as “rural area” were suicides committed in the remaining non-urban areas (area: 776.7 km^2^, population: 84,873). In addition, we also analysed in detail suicide methods as well as the impact of alcohol abuse on suicide rates. Causes of death were classified according to the 10th revision of the International Statistical Classification of Diseases and Related Health Problems (ICD-10).

The statistical analyses were performed with the statistical data analysis software system STATISTICA, version 13 (StatSoft, Tulsa, Oklahoma, USA). Parametric statistical procedures (t-Student test and ANOVA) were performed to test for significant statistical differences between study groups. The *χ*^2^ test was used to detect the possible differences between analysed groups with respect to sex, place and season of the year at the time of death, and the method of suicide. The Pearson correlation coefficient (*r*) was used to assess possible correlations between studied numerical variables (number of suicides and age of deceased). *P* values < 0.05 were considered as statistically significant.

## Results

### Number of suicides

**Fig. 1:**
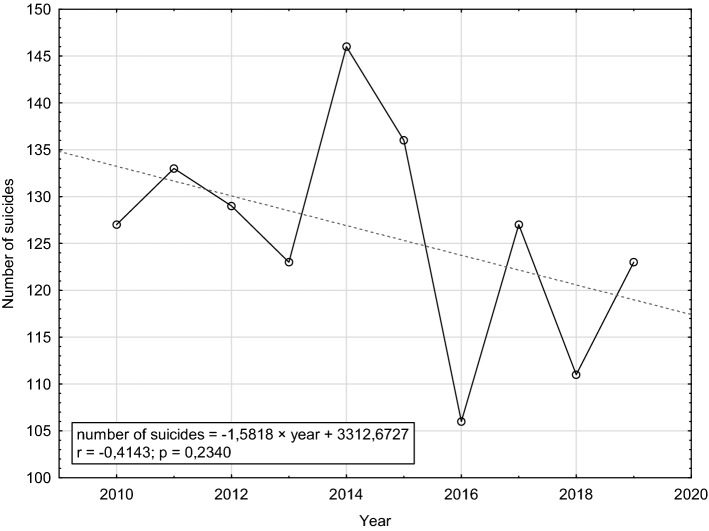
Numbers of suicides in the Tri-City metropolitan area in 2010–2019

In the analysed years 2010–2019, there were 1,261 suicides in the Tri-City metropolitan area, which corresponds to the annual suicide rate of 14.6 per 100,000 residents. Despite a marked decreasing trend of 1.5 per year, in the analysed period we observed a sharp increase in the number of suicides, from 123 in 2013 to 146 in 2014, with a subsequent drop to 106 in 2016 (Fig. 1).

**Fig. 2:**
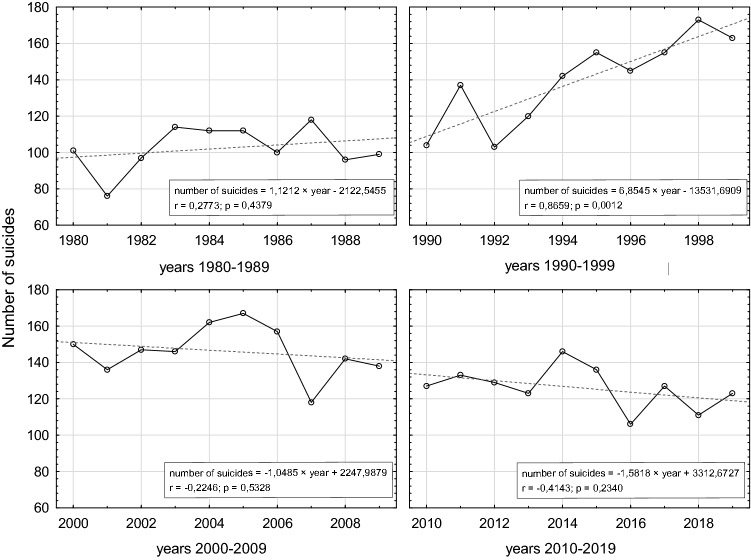
Comparison of the dynamics of changes in the number of suicides in the Tri-City metropolitan area in the following decades

A comparison of the dynamics of changes in the number of suicides in 2010–2019 with preceding time interval (1980–2009) [14] indicates the continuation of a downward trend since the beginning of twenty-first century, more strongly accentuated in 2010–2019 (Fig. 2).

In the analysed material, the mean monthly number of suicides was 105, with the highest value in November (120) and the lowest one in February (105). Moreover, a correlation was observed between the number of suicides and the season of the year, with the highest number of suicides in autumn (337) and the lowest in winter (295) [*χ*^2^ = 40.72971; df = 27; *p* = 0.044].

### Sex and age of suicides

**Fig. 3:**
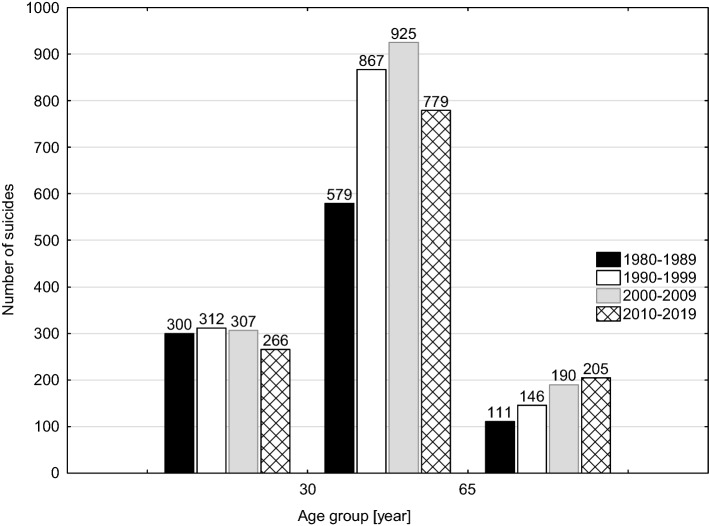
Changes in the numbers of suicides in three age groups (0–30; 30–65; 65 +) in 1980–2019

In the analysed cohort, male suicides constituted 79.6% (*n* = 1004) and female suicides constituted 20.3% (*n* = 256). A comparison between our current and previous results indicates that the proportion of male suicides in the total group increased, however insignificantly, from 76.7% in the years 1980–1989 through 77.9% in the years 1990–1999 to 79.7% in the years 2010–2019 (*p* = 0.42). The mean age of suicides was 47.6 years (range 12–95; SD 18.3), and was the highest if we compare the four consecutive decades within the years 1980–2019: 42.1 years in 1980–1989; 43.7 years in 1990–1999; 46.2 years 2000–2009; 47.6 years 2010–2019 (*p* < 0.001). Simultaneously, a decreasing trend in the mean age of female suicides was observed in the last decade. Therefore, the means of age in male and female subgroups in this time interval were comparable: 47.2 and 48.8 years of age, respectively. The increase in mean age of suicides observed currently was a consequence of the increased number of oldest suicide victims (65 +) paralleled by a decrease in the youngest subgroup (Fig. 3).

**Fig. 4:**
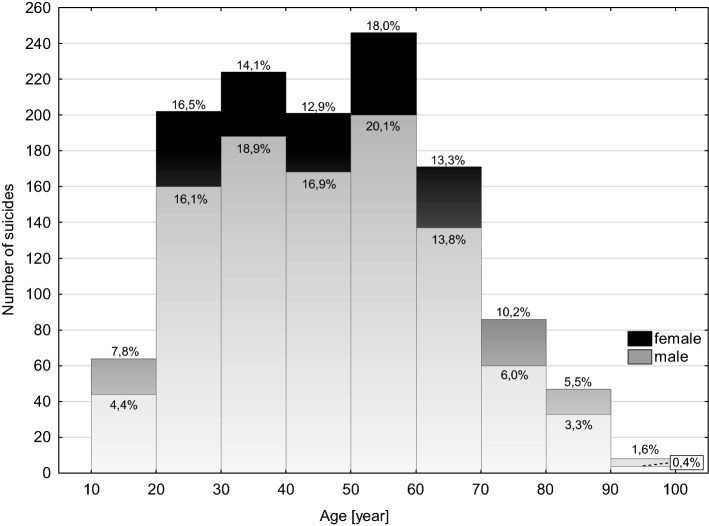
Histogram of the age of suicides according to sex (percentages calculated separately for males and females)

The most numerous groups of suicides were victims aged 50–60 (*n* = 226), followed by those aged 30–40 (*n* = 224). Percentage-wise, suicide completers aged 30–70 were predominantly male, while females tended to predominate in the youngest and oldest subgroups, i.e. 10–30 and 70–100 years (Fig. 4).

### Place of death

**Fig. 5:**
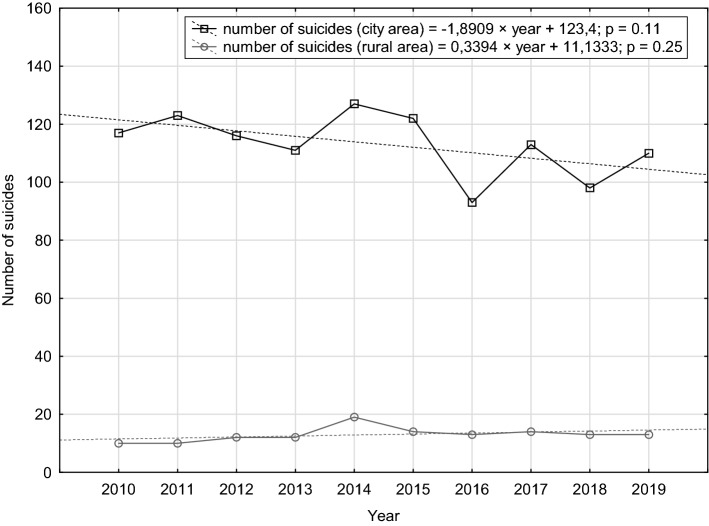
Comparison of the dynamics of changes in the number of suicides in urban and rural areas

In the analysed cohort, 1130 (89.7%) suicides were committed in the urban area and 130 (10.3%) in the rural area. The annual suicide rate in the urban and rural areas was 14.5 per 100,000 and 15.3 per 100,000, respectively (*p* = 0.82). Different tendencies were observed in those regions: in the urban area a decreasing trend was observed, while in the rural area the number of suicides increased (Fig. 5). The latter tendency was accompanied by an increased percentage of suicides in the rural area in the total number of suicides (from around 8.5% in 2010 to over 12.0% in 2019).

In the study group, the number of suicides among women in urban area was almost twice as high as in rural area, which was accompanied by an increased percentage of male suicides in the former (87.7%) compared to the latter area (78.7%) [*χ*^2^ = 5.764662; df = 1; *p* = 0.01635]. The mean age of suicide victims in the rural area was significantly lower compared to the urban area (44.3 vs. 47.9 years of age, respectively; *t* =  − 2.13726, *p* = 0.032771).

### Suicide method

In the studied cohort, the most common methods of suicide were: hanging (65.27%), jumping from height (13.48%) and self-poisoning (8.41%). The methods such as poisoning, jumping from height and drowning were statistically more frequent in females compared to males, whereas hanging and firearm injury were less frequent in this subgroup (*χ*^2^ = 113.0769; df = 7; *p* = 0.00001). On the other hand, suicides by jumping from height and jumping in front of moving vehicle were observed less frequently in the rural areas, where hanging was more frequent compared to the urban areas—Table 1 (*χ*^2^ = 27.2942; df = 7; *p* = 0.00029).

**Table 1:** Methods of suicide—total number and by sex and place of death

	Total	Sex		Place of death	
		Female	Male	Rural area	Urban area
Hanging	823	115 (44.92%)	708 (70.52%)	105 (80.77%)	717 (63.45%)
Jumping from a high place	170	58 (22.66%)	112 (11.16%)	2 (1.54%)	168 (14.87%)
Self-poisoning	106	48 (18.75%)	57 (5.68%)	10 (7.69%)	96 (8.50%)
Jumping in front of moving object (vehicle)	75	16 (6.25%)	59 (5.88%)	3 (2.31%)	72 (6.37%)
Sharp object	30	3 (1.17%)	27 (2.69%)	3 (2.31%)	27 (2.39%)
Firearm discharge	27	0 (0.00%)	27 (2.69%)	3 (2.31%)	24 (2.12%)
Drowning and submersion	18	11 (4.30%)	7 (0.70%)	1 (0.77%)	17 (1.50%)
Other methods	12	5 (1.95%)	7 (0.70%)	3 (2.31%)	9 (0.80%)

**Fig. 6:**
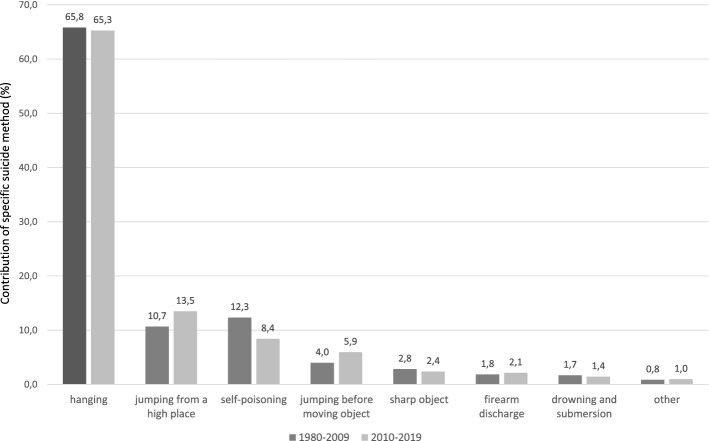
Comparison of the frequency of methods of suicide committed in 1980–2009 and 2010–2019

A comparison of the frequency of specific suicide methods in the analysed years (2010–2019) with the previous period (1980–2009) shows a clear increase in recent years in such suicide methods as jumping from height and jumping in front of moving object, and a decrease in self-poisoning in the years 2010–2019 (Fig. 6).

In the currently analysed time interval, the highest mean age was observed in suicide victims who died from jumping from height (50.8 years) or sharp injuries (52.0 years). In opposite, jumping in front of moving object was most frequently observed in the youngest victims (37.2 years on average).

### Alcohol inebriation

In the analysed cohort of suicide victims, the blood alcohol concentration (BAC) was assayed in 1171 cases and in 528 cases (45.1%) BAC was equal or higher than 0.2‰. The mean BAC in the entire cohort was 0.79‰ [SD 1.08‰; range 0.0–4.7‰], and in the group of inebriated suicides it was 1.75‰. The mean BAC in males was 0.87‰ and thus it was almost twice as high as the mean BAC in females (0.46‰). On the other hand, when analysing only the inebriated cases, the BAC values in males and females were comparable (approx. 1.75‰ on average in both subgroups). The mean age of inebriated suicides was lower compared to the remaining cases (44.0 vs. 50.1 years of age, respectively; *t* =  − 5.68230, df = 1160, *p* < 0.01). No significant difference in the mean BAC values was observed between suicides in urban and rural areas. Similarly, no significant difference was found in the percentage of inebriated suicides between urban and rural areas (44.7% and 47.6%, respectively). Inebriated victims outnumbered the remaining cases only in the subgroup of hanging (51.4%), while in other subgroups they were less frequent, which was accentuated in the subgroups: jumping from height (25.3%), jumping in front of moving object (34.3%), and sharp object injuries (36.0%).

## Discussion

### Number of suicides

In 2020, more than twice as many people committed suicide in Poland (4553) than died in road accidents despite the overall decline in cases of unnatural deaths. Moreover, in the years 1980–2010 we observed a growing proportion of suicides in that group of deaths, from 16.7 to 26.8%, with a subsequent drop to 22.4% in 2020 [15]. The highest suicide rates are recorded in Central and Eastern Europe, in particular in Lithuania (26.1 per 100,000) and Latvia (20.1 per 100,000) [2]. The WHO states that in Poland, in 2019, the crude suicide rate was 11.3 per 100,000, which was less than the rate calculated for the agglomeration of the Tri-City based on this study (14.6 per 100,000). The authors of current study cannot answer the question whether these differences are related to the specifics of the study region (a large urban area where psychopathological and socioeconomic factors may be more accentuated as compared to other regions) or to hypothetically inaccurate reporting of data by official authorities. The latter view could be supported by differences in the number of suicides reported by the National Police Headquarters (NPH) and Statistics Poland (SP), e.g., in 2012, the number of suicides published by the NPH and SP was 4177 and 6365, respectively. Perhaps for that reason, the WHO ranks the quality of Polish data as “medium” on a three-degree scale and their usability between 68 and 72% [16].

The decreasing trend in the number of suicides observed in the years 2010–2019 corresponds to global trends, which started at the end of the previous century and are mainly connected with the implementation of preventive measures since the 1950s [2, 17]. In the area of the Tri-City agglomeration, this tendency became particularly evident at the beginning of the twenty-first century after a sharp increase in the number of suicides in the last decade of twentieth century. This increase could be partially explained by the abrupt change of political system in Poland after the fall of communism. In accordance with Durkheim’s theory, a period of “anomie” could be a consequence of political turbulence, described in Poland as the “trauma of the great change”. The sharp increase in the number of suicides in the years 1990–1999, 6 times higher than in the years 1980–1989, is believed to be the outcome of the changes and social cost of the system transformation after 1989 [18, 19]. Finland, which in the second half of the twentieth century recorded exceptionally high suicide rates, is a model example of the effective implementation of suicide preventive measures. In Finland, special attention was paid to the thorough examination of the scale of the problem, including the allocation of very high funds to suicide research, as a result of which Finnish researchers published over 500 papers on suicidology in the years 1989–2018 [20]. Then, on the basis of large-scale so-called “psychological autopsy”, it was proven that 93% of suicides could be diagnosed with at least one mental disorder, most often depression and alcohol abuse or addiction [21]. That, in turn, allowed the researchers to understand the complex nature of suicide and indicate one of the major causes for the high suicide rates, which was the “diagnostic weakness” of the health care system in terms of mental diseases diagnostics and treatment. In the years 1990–2015, the suicide rate in Finland decreased from 26.96 per 100,000 to 12.62 per 100,000. In the same time, the number of suicides in Poland, instead of decreasing, grew slightly (from 13.57 to 13.95 per 100,000).

### Sex and age of suicides

The problem of suicide is particularly accentuated in males, for whom, based on the official data from the Statistics Poland (SP), the suicide rate in 2020 was 21 per 100,000, while, at the same time, the female suicide rate was almost 7 times lower at 3.1 per 100,000. In the analysed cohort, there were also more numerous male cases, who accounted for almost 80% of studied cases. Male-to-female suicide ratios above 3 are specific for high-income countries, i.e., countries where socioeconomic factors such as unemployment and wage level have a significant impact on suicide. They are particularly accentuated in professionally active males, i.e. the social group most affected by the stress of having to support financially a family [2, 13, 22]. The aforementioned social and economic factors (unemployment/loss of work, low wages) may predispose to suicidal behaviour—in turn, a suicide death in the family can result in the worsening of the financial situation and thus can increase poverty [5, 23, 24]. The aforementioned relationship between economic factors and suicide—of a vicious circle character—is believed to be a consequence of two important factors: (1) adverse economic factors (recession) can contribute to the reduction of per capita income, and (2) a reduction in state tax revenues can in turn hinder access to health services, including mental health services [13]. Notably, in difficult financial situation, for people with mental disorders and older people may be difficult to find and retain a job, which can be another stressful factor. Considering the fact that men are reluctant to discuss their problems and are more likely to hide their emotional pain rather than seek help, it seems understandable why men are at a higher risk of suicide [22]. It is worth pointing out that the level of funding for psychiatry in Poland (in terms of psychiatric care, among them the treatment of addictions), regardless the economic situation, is extremely low and constitutes just 3.04% of the budget of the National Health Fund, in comparison with 6–8% in Western European countries. Due to the inadequate number of psychiatrists, including child psychiatrists, and the resulting prolonged appointment waiting time (3.6 months on average), one of the basic suicide preventive measures, i.e., fast psychiatric diagnosis, is inefficient. Moreover, the “National Mental Health Protection Programme”, which was implemented in 2010, has failed, which was described in detail in the report of the Supreme Audit Office in 2017. One of the major objections of auditors was the incorrect organisation and low funding of psychiatric care in Poland.

Suicide, as one of three main types of violent death, next to homicides and accidents, is a major cause of premature mortality [25]. In the currently studied cohort, the average age of suicide victims was 47.6 years, clearly lower than the SP’s reported life expectancy of 74.1 for men and 81.1 for women in 2019. In the analysed cohort, middle-aged suicides formed the largest group. Importantly, this subgroup was predominantly male, which was related to the difference in mean age between males and females in the entire cohort (in males approx. 1.5 years lower). Therefore, our results do not confirm currently presented concept of the increasing proportion of the youngest suicide victims (< 35) in the entire suicide cohort [2, 5, 26]. On the contrary, we observed a decreasing trend in that age group. Simultaneously, an increase in suicides of the oldest people (65 +) was found, thus the analysed cohort of suicides has “aged” in the past 40 years. The WHO reports revealed that similar patterns are also found in the USA, Australia and many EU countries, which may be a consequence of population ageing. However, there are clear regional differences in the mean age of suicide cohorts, as the majority of suicides committed by young individuals (88%) occurs in low and middle-income countries [2], where social inequalities and the poor health care play major roles [27]. Since 2009, Poland has been classified as a high-income country.

### Suicide method and place of death

Given the official data of the Polish Statistical Office, suicide rates have been observed to be much higher in rural areas (around 15 per 100,000) than in urban areas (around 10 per 100,000) for years. In the analysed area of the Tri-City agglomeration, differences in suicide rates between the urban area (14.5 per 100,000) and the rural area (15.3 per 100,000), albeit not as pronounced, were also observed, which were accompanied by an increasing number of suicides in the rural area, contrary to the urban area, where a decreasing trend was observed. Other reports confirm that the increasing proportion of suicides in rural areas is a global problem [25]. There are many factors, which play a role: an easy access to pesticides as one of the most lethal suicide methods, low education level, low access to health care, including mental health services, as well as unemployment and low standard of living. These factors, when put into the perspective of the whole country, reflect the image of particular social inequality between rural and urban areas [28]. The social inequalities are also manifested in biological differences between youth in rural and urban areas, such as body height, weight, mean age of menarche, etc., which is manifested in the perception of one’s self as inferior [28]. Men are particularly affected by the inferior self-perception, which may explain why suicides in rural areas occur so often among younger males.

The official data indicate that hanging is the most frequent suicide method in Eastern European countries [11] and, together with firearm suicides and pesticide poisoning, is responsible for the majority of suicides worldwide [2]. In the analysed material, hanging (65.3%), jumping from height (13.5%) and self-poisoning (8.4%) were found to be the most common methods of suicide. Hanging is a highly accessible and lethal suicide method and thus this method is very common. In turn, a high incidence of jumping from height might be associated with the presence of high buildings in the agglomeration of the Tri-City, including whole districts with 10-floor blocks of flats built in the 1960s and 1970s. On the other hand, the low proportion of self-poisoning in the analysed cohort might be caused by diverse factors. Among them are: (1) a small proportion of suicides in the rural area where pesticide poisoning is the predominant suicide method, at least from global perspective [2, 29], and (2) a probable underestimation of poisoning as so-called “soft” (non-violent) suicide method, which might be recognised as a natural death (i.e. disease related) without subsequent autopsy including toxicological assay. The higher proportion of so-called “soft” suicide methods in females (especially poisoning) may be related to the tendency to choose “less painful” and “less traumatic” methods [11]. On the contrary, however, a twofold higher proportion of jumping from height in females compared to males suggests a high level of self-aggression and determination in suicidal act in a subgroup of female victims. The availability of high buildings in the Tri-City (as one of risk factors) may play an important role, which is also suggested by a small number of suicides by jumping from height in the rural area (1.54%, *n* = 2).

A comparison between our current (2010–2019) and previous results (1980–2009) suggests an increased proportion of violent methods such as jumping from height and jumping in front of moving object, which are associated with high level of self-aggression and impulsiveness as important behavioural endophenotypes in the aetiology of suicide-related behaviours [30].

### Alcohol inebriation

Alcohol inebriation is a significant risk factor of suicide-related behaviours and, in the opinion of some authors, even more important than alcohol abuse or dependence [31], currently defined as alcohol use disorders. It was reported that up to 66% of suicides are inebriated at the time of death [32]. The current study, considering the results of our previous research [14], shows an increase in proportion of intoxicated suicides from 40.0% in the years 2000–2009 to 45.1% in the years 2010–2019. The mean age of intoxicated suicides was approx. 6 years lower (44.0 years) than remaining victims (50.1 years), which accentuates the negative impact of alcohol abuse. Simultaneously, the low proportion of inebriated victims in such subgroups as jumping from height (25.3%) and jumping in front of moving object (34.3%) suggests other risk factors, in particular the previously mentioned behavioural endophenotypes of self-aggression and impulsiveness.

### Seasonality

The current study corresponds with previous reports on the relationship between the number of suicides and the season (“Seasonality”). The highest number of suicides in autumn (November) found in the cohort and the sharp increase in suicides in early spring suggest an involvement of serotonergic system disorders in the observed phenomenon [33], as well as other factors, including day-night cycle, and melatonin and cortisol activity.

### Limitations

We do not know whether the suicide victims with elevated blood alcohol levels also had harmful alcohol use or addiction. Unlike in psychiatric clinics, our department does not routinely determine markers for alcohol-related liver damage.

The extended toxicological analysis is also not routinely performed at our department because the supervising prosecutors order this analysis in rare cases. As a result, a complete evaluation of the impact of toxic substances on suicide epidemiology in the studied region of Poland could not be made.

## Conclusions

1. In the analysed years 2010–2019, in the Tri-City and the adjacent Gdańsk County 1,261 suicides were recorded, with an average annual suicide rate of 14.6 per 100,000.
2. In the analysed period, a decreasing trend in the number of suicides was observed, however the number of suicides in rural areas (Gdańsk County) increased.
3. Suicides were committed most often by middle-aged males. At the same time an increasing number of suicides was observed in the group of the oldest persons (65 +). Therefore, our data do not correspond with reports accentuating the increasing proportion of youngest suicide victims.
4. In the studied cohort of suicides, a clear influence of alcohol abuse on the suicide phenomenon was observed, especially in the younger subgroup of victims.

## Data Availability

On behalf of all authors, the corresponding author states that the data being reported are accurate and are coming from the official source.
